# Lipoprotein(a) and Atrial Fibrillation: A Systematic Review and Meta-Analysis

**DOI:** 10.3390/jcm14217770

**Published:** 2025-11-01

**Authors:** Bartosz Maj, Michal Pruc, Pawel Czubak, Iga Romanska, Karol Momot, Marta Klos, Kamil Krauz, Aleksandra Mielnik, Zbigniew Siudak, Katarzyna Kotfis, Lukasz Szarpak

**Affiliations:** 1 Department of Anesthesiology and Intensive Care, Health Center of Tomaszow Mazowiecki, 97-200 Tomaszow Mazowiecki, Poland; 2 Department of Clinical Research and Development, LUX MED Group, 02-678 Warsaw, Poland; 3 Institute of Biological Sciences, The John Paul II Catholic University of Lublin, 20-708 Lublin, Poland; 4 Students Research Club, Collegium Medicum, The John Paul II Catholic University of Lublin, 20-708 Lublin, Poland; 5 Chair and Department of Experimental and Clinical Physiology, Laboratory of Centre for Preclinical Research, Medical University of Warsaw, 02-097 Warsaw, Polandkamilkrauz01@gmail.com (K.K.); 6 3rd Department of Internal Diseases and Cardiology, Międzylesie Specialist Hospital in Warsaw, Medical University of Warsaw, 04-749 Warsaw, Poland; 7 Institute of Medical Biology, The John Paul II Catholic University of Lublin, 20-708 Lublin, Poland; 8 Doctoral School, Medical University of Warsaw, 02-097 Warsaw, Poland; 9 Department of Primary Care, LUX MED Fieldorfa Oncology Hospital, 04-125 Warsaw, Poland; olamielnik99@gmail.com; 10 Collegium Medicum, Jan Kochanowski University, 25-317 Kielce, Poland; 11 Department of Anesthesiology, Intensive Care and Pain Management, Pomeranian Medical University, 70-204 Szczecin, Poland; katarzyna.kotfis@pum.edu.pl; 12 Technology Transfer Center, The John Paul II Catholic University of Lublin, 20-950 Lublin, Poland; 13 Henry JN Taub Department of Emergency Medicine, Baylor College of Medicine, Houston, TX 77030, USA

**Keywords:** lipoprotein(a), atrial fibrillation, biomarkers, systematic review, meta-analysis, cardiovascular risk, inflammation

## Abstract

**Background/Objectives:** The most prevalent prolonged cardiac arrhythmia and a significant global health burden is atrial fibrillation (AF). Although its connection to AF is still unknown, lipoprotein(a) (Lp(a)), a genetically determined lipoprotein with pro-inflammatory and pro-atherogenic characteristics, has been linked to cardiovascular disease. The purpose of this study was to measure and assess the relationship between circulating Lp(a) levels and AF. **Methods**: In compliance with the PRISMA 2020 guidelines, a systematic review and meta-analysis were carried out using a protocol that was preregistered in PROSPERO (CRD420251153244). Comprehensive searches of PubMed/MEDLINE, Embase, Web of Science, Scopus, the Cochrane Library, and Google Scholar up until September 2025 were used to find observational studies comparing circulating Lp(a) levels in adults with and without AF. **Results**: Circulating Lp(a) concentrations were significantly higher in AF patients than in controls across 10 studies (pooled MD = 2.81; 95%CI: 1.58–4.05; *p* < 0.0001). In the subgroup analysis by geographical setting, studies conducted in Asia and studies from Europe/USA exhibited a statistically significant effect. Despite the extreme heterogeneity (I^2^ = 99%), sensitivity analyses verified that the overall effect was stable. **Conclusions**: Our pooled analysis revealed a statistically significant association between Lp(a) and AF; however, the certainty of the evidence was rated as very low according to the GRADE methodology. To elucidate causality, enhance risk stratification, and investigate whether Lp(a)-lowering tactics could alter AF risk, large, prospective, multi-ethnic studies with standardized biomarker assessment are needed.

## 1. Introduction

Atrial fibrillation (AF) is the most common sustained cardiac arrhythmia and an escalating public health problem worldwide. Its prevalence continues to rise, largely because populations are aging and more people are living longer with coronary artery disease and heart failure. AF is much more than just a cardiac rhythm problem; it greatly raises the chances of complications, including ischemic stroke, heart failure, cognitive decline, and early death, which poses an increased burden on healthcare facilities with enormous costs and significant effect on the daily lives of patients [1,2]. Classical risk factors such as advanced age, hypertension, diabetes, obesity, and structural heart disease account for a significant portion of this burden; however, a considerable amount of risk remains unexplained [3]. These findings have heightened endeavors to discover novel biomarkers that enhance risk prediction and elucidate the mechanisms of atrial arrhythmogenesis.

Among diverse lipid-related biomarkers under investigation, lipoprotein(a) (Lp(a)) has attracted growing attention and has emerged as a compelling candidate. Lp(a) is a genetically determined lipoprotein composed of an LDL-like particle covalently linked to apolipoprotein(a) [4]. Elevated circulating Lp(a) levels are independent, causal determinants of atherosclerotic cardiovascular disease (most notably myocardial infarction and calcific aortic valve stenosis) [5,6]. Its pathogenicity reflects a combination of pro-atherogenic and pro-thrombotic actions, including cholesterol deposition within the arterial wall, impaired fibrinolysis, and amplification of vascular inflammation [7]. A connection between Lp(a) and AF is biologically plausible yet remains inadequately characterized. The pro-inflammatory and pro-oxidative characteristics of Lp(a), including the transport of oxidized phospholipids and the recruitment of inflammatory cells, may promote atrial fibrosis and structural remodeling, establishing a substrate for the initiation and maintenance of AF. The strong association of Lp(a) with aortic valve stenosis is also relevant, as valvular disease is a recognized driver of AF; indirect effects on cardiac structure could therefore contribute to risk [8].

Epidemiologic findings remain mixed. Several large cohort and case–control studies report that higher Lp(a) concentrations are associated with incident AF [9], whereas others find no relationship [10,11] or even suggest an inverse association, with lower Lp(a) linked to higher AF risk [12,13]. These discrepancies likely reflect differences in study design, population characteristics, sample size, assay techniques, and adjustment for confounding factors.

AF has recently gained recognition as a clinical manifestation of atrial cardiomyopathy, a structural and functional disorder of the atrial myocardium that facilitates arrhythmogenesis and thrombogenesis [14]. This conceptual framework underscores the significance of inflammation, fibrosis, and atrial remodeling as principal mechanisms contributing to AF. Lp(a) has not yet been implicated in the etiology of atrial cardiomyopathy; however, its pro-inflammatory and pro-fibrotic characteristics may potentially influence these pathogenic mechanisms. Consequently, investigating the correlation between Lp(a) and AF may yield innovative insights into the inflammatory aspect of atrial cardiomyopathy.

Although the role of Lp(a) in cardiovascular disease is well established, its contribution to atrial fibrillation remains uncertain, making clarification of this association a matter of considerable scientific and clinical importance. We therefore conducted a systematic review and meta-analysis comparing circulating Lp(a) concentrations in individuals with AF versus non-AF controls, with two aims: to provide a more precise pooled estimate of the association and to explore sources of heterogeneity that can guide future research.

## 2. Materials and Methods

### 2.1. Study Design and Registration

This study was conducted as a systematic review and meta-analysis in accordance with the PRISMA 2020 (Preferred Reporting Items for Systematic Reviews and Meta-Analyses) statement [15]. The study protocol was developed a priori and registered in the International Prospective Register of Systematic Reviews (PROSPERO; registration no. CRD420251153244) to ensure methodological transparency and reduce the risk of selective reporting (Supplementary Table S1). The design followed established Cochrane methodology, incorporating predefined objectives, eligibility criteria, and statistical strategies. By following these standards, we tried to put together a complete and fair summary of the evidence that was available, while also pointing out important gaps and limitations in what is known about the subject.

### 2.2. Eligibility Criteria

Inclusion criteria were defined using the PECOS framework. We included studies enrolling adult patients (≥18 years) with atrial fibrillation (Population) in whom circulating lipoprotein(a) concentrations were measured (Exposure) and compared with individuals without atrial fibrillation from the same or comparable populations (Comparator). Eligible studies had to report the difference in mean Lp(a) levels between AF and non-AF groups, or provide sufficient data to calculate mean differences with 95% confidence intervals (Outcome). We restricted inclusion to observational study designs, including prospective and retrospective cohorts, case–control investigations, and cross-sectional analyses, published as full-text articles in peer-reviewed journals (Study design).

Studies were excluded if they lacked a control group, did not report Lp(a) measurements, or were limited to case reports, reviews, editorials, or conference abstracts without sufficient data. Experimental studies conducted in animals or in vitro investigations were excluded. In cases where multiple publications originated from the same cohort, we retained the most comprehensive and recent dataset. Only peer-reviewed articles were considered for inclusion.

### 2.3. Search Strategy

A comprehensive literature search was conducted in PubMed/MEDLINE, Embase, Web of Science, Scopus, the Cochrane Library, and Google Scholar from database inception until 15 September 2025. The search strategy combined controlled vocabulary (e.g., MeSH and Emtree terms) and free-text keywords. Examples of search terms included: “lipoprotein(a)”, “Lp(a)”, “Lipoprotein a” combined with “atrial fibrillation” or “AF”.

Only studies published in English were considered eligible. References of included articles and relevant reviews were manually screened to identify additional eligible studies. All retrieved records were managed using reference management software, and duplicates were removed prior to screening. Two reviewers independently screened titles, abstracts, and full texts for eligibility. The full search strategy for each database is provided in Supplementary Table S2.

### 2.4. Study Selection

All references identified through the database searches were first imported into a reference management system, and duplicate records were removed. The selection process was then carried out in two steps. Initially, two reviewers independently screened the titles and abstracts to discard papers that were clearly irrelevant, while keeping those that seemed to meet the broad inclusion criteria. Full texts were subsequently obtained for the remaining articles and examined carefully with respect to study design, characteristics of the study population, and the availability of data on Lp(a) and AF.

When the investigators disagreed on the eligibility of a particular study, the matter was discussed until a consensus was reached. If agreement could not be achieved, a third member of the team reviewed the article and made the final decision. The goal of this layered approach was to lower the risk of mistakes and make sure that the final set of studies included the best evidence for analysis.

### 2.5. Data Extraction

Data from all studies that fulfilled the inclusion criteria were gathered using a structured template that had been agreed upon in advance. Two investigators worked independently throughout the process. For each paper we noted the main bibliographic details (author, year of publication, country, study type) together with the size and characteristics of the study population, including the number of atrial fibrillation cases and controls, age and sex distribution, and, where reported, relevant comorbidities. Particular attention was given to how lipoprotein(a) was measured, documenting the laboratory technique, reporting units, and whether the results were provided against standardized reference ranges.

The primary outcomes extracted were mean levels of Lp(a) with their corresponding measures of variation (standard deviation, standard error, interquartile range, or 95% confidence intervals). If only medians and ranges were available, these were converted into approximate means and standard deviations using established statistical formulas. Whenever studies reported effect sizes directly, such as mean differences with confidence intervals, odds ratios, hazard ratios, or relative risks, these were also recorded together with any covariates used in adjusted analyses.

All studies furnished comprehensive numerical data essential for the analysis; consequently, further communication with the original study authors was unnecessary, and no data were derived from graphical representations. Standard formulas were used to make sure that the results were the same across studies when they were reported in different statistical formats.

### 2.6. Risk of Bias Assessment

The ROBINS-E tool (Risk of Bias in Non-randomized Studies of Exposures), which is intended to evaluate observational evidence in cases where exposures rather than interventions are being studied, was used to assess the risk of bias for non-randomized studies [16]. Each study was evaluated independently by two investigators in seven areas: (i) confounding; (ii) participant selection; (iii) exposure measurement and classification; (iv) deviations from intended exposures; (v) missing data; (vi) outcome measurement; and (vii) selection of the reported result.

The risk of bias was rated as low, moderate, serious, or critical for each domain. The highest level of bias across all domains was taken into account when making overall judgments. Reviewers’ disagreements were settled by consensus and discussion, with a third investigator mediating if needed. Confounding (e.g., controlling for age, sex, comorbidities, and cardiovascular risk factors) and exposure measurement (e.g., laboratory assays of Lp(a) concentrations and HDL functional parameters) were given special consideration because the included studies were primarily observational cohorts and case–control designs.

### 2.7. Certainty of Evidence Assessment

The GRADE (Grading of Recommendations, Assessment, Development, and Evaluation) approach was used to figure out how sure the evidence was for each outcome. It looks at the overall confidence in effect estimates across five areas: (i) risk of bias, (ii) inconsistency, (iii) indirectness, (iv) imprecision, and (v) publication bias/other considerations [17]. For each outcome, the evidence was classified as having high, moderate, low, or very low certainty.

### 2.8. Statistical Analysis

Review Manager software (RevMan, Version 5.4, The Cochrane Collaboration, Copenhagen, Denmark) was used to analyze the data. The meta and metafor packages in R (version 4.5.1; R Foundation for Statistical Computing, Vienna, Austria) and the statsmodels (v0.14), scipy (v1.11), numpy (v1.26), and matplotlib (v3.7) libraries in Python (version 3.11) were also used to perform complementary and diagnostic analyses (sensitivity analyses, meta-regression, publication bias tests, and graphical outputs). Statistical significance was defined as a two-sided *p*-value < 0.05.

Cochrane guidelines were followed when performing statistical analyses. Mean differences (MDs) with associated 95% CIs were used to represent study-level effect sizes. For all pooled analyses, we used a random-effects model (DerSimonian–Laird approach) that completely re-estimated the between-study variance (τ^2^). Cochran’s Q statistic, the I^2^ statistic (which indicates the percentage of overall variability attributable to between-study heterogeneity), and the estimate of τ^2^ were used to quantify statistical heterogeneity [18,19]. In order to account for the anticipated range of effects in future settings, prediction intervals were also computed.

In a leave-one-out (LOO) sensitivity analysis, we re-estimated the random-effects model after sequentially excluding each study to assess the robustness of the pooled estimates. We measured the change in the pooled estimate compared to the full model and recorded the pooled effect size, 95% CI, and heterogeneity indices (Q, I^2^, and τ^2^) for each iteration. Furthermore, a Baujat plot was created to show how each study affected the pooled mean difference (absolute change, *y*-axis) and contributed to the overall heterogeneity (component of Q, *x*-axis). These supplementary resources offer an open assessment of the reliability of the results and the impact of separate research.

We performed a number of random-effects meta-regression analyses to investigate the sources of heterogeneity in more detail. Prespecified moderators were chosen based on study features and biological plausibility: (i) the type of biomarker (Lp(a) versus HDL function/oxidation); and (ii) the clinical population (CAD, HF, or other/unspecified). Additionally, a combined model with both moderators was fitted. Weights were defined as wᵢ = 1/(vᵢ + τ^2^), where vᵢ is the within-study variance, and τ^2^ was re-estimated in all models. Using the t distribution with k–p degrees of freedom (where k is the number of studies and *p* is the number of model parameters), statistical inference was based on Hartung–Knapp adjustments.

By visually examining funnel plots, publication bias and small-study effects were evaluated. Egger’s regression test and Begg’s rank correlation test were used for formal testing. In order to assess the robustness of statistical significance, we also computed Rosenthal’s fail-safe N and used the Duval and Tweedie trim-and-fill method to estimate a bias-adjusted pooled effect. Although their limited power under high heterogeneity conditions was acknowledged, these analyses were only carried out because the number of included studies (k = 10) met the minimal threshold (≥10) suggested by the Cochrane Handbook [20].

## 3. Results

### 3.1. Search Results Summary

**Figure 1:**
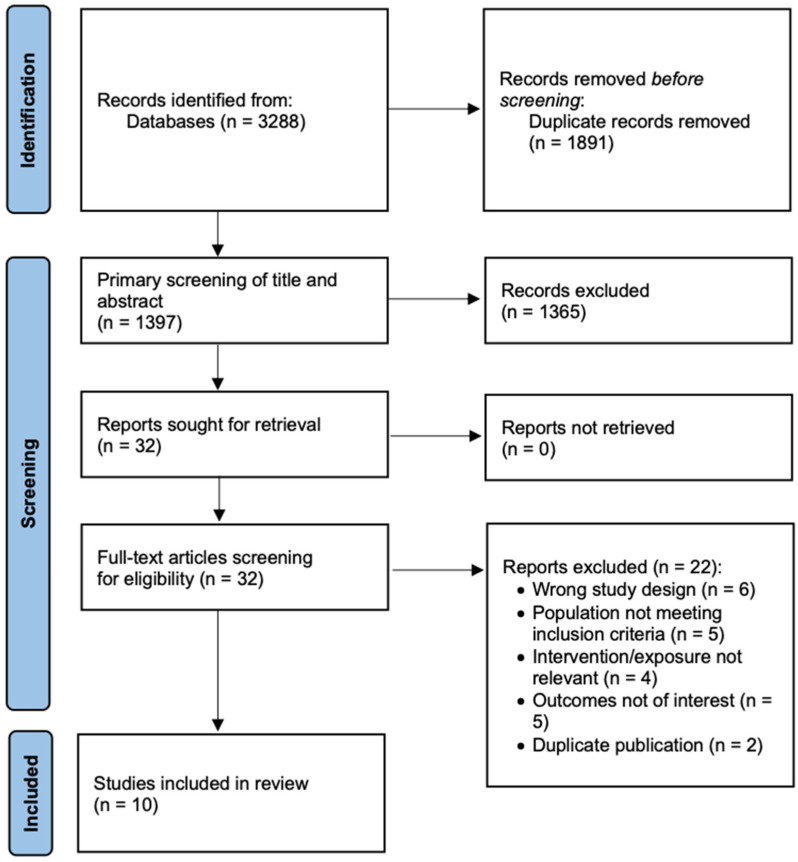
PRISMA 2020 flow diagram of the systematic literature search and study selection process.

A total of 3288 records were retrieved through systematic searches of electronic databases (PubMed, Embase, Web of Science, Cochrane Library, and Google Scholar), complemented by manual screening of reference lists. After removal of duplicates, 1397 unique records were subjected to title and abstract screening. Of these, 1365 articles were excluded for not meeting the prespecified inclusion criteria (e.g., irrelevant population, intervention, comparator, outcome, or study design). The full texts of 32 potentially eligible articles were then assessed in detail, leading to the exclusion of 22 studies due to predefined reasons such as insufficient data for effect size calculation, overlapping study populations, or non-human research. Ultimately, 10 studies fulfilled all eligibility criteria and were included in the meta-analysis [9,11,21,22,23,24,25,26,27,28,29]. The complete study selection process is summarized in Figure 1.

### 3.2. Characteristics of the Included Studies

This meta-analysis incorporated ten studies published between 2006 and 2025, encompassing a total of 32,473 individuals with atrial fibrillation and 377,430 participants in the control groups. With individual sample sizes ranging from 178 to 365,899, the body of evidence was methodologically diverse and included case–control investigations, retrospective cohorts, and prospective cohort studies. This variation in study design reflects both the range of available data and the methodological heterogeneity that could affect effect estimates.

According to geography, the majority of research was carried out in China [9,22,23,24,27,28], indicating a preponderance of Asian cohorts, while other contributions came from Europe [25,27,29] and the United States [11], providing some geographical diversity. Table 1 presents a comprehensive summary of individual study characteristics.

The quality of the included studies was evaluated with the ROBINS-E tool, which is specifically designed for observational research on exposures. Among the ten studies, two (Jiang et al. [22] and Mora et al. [11]) were considered to have a low risk of bias, while one (Li et al. [25]) was judged as low to moderate. The remaining papers were classified as having a moderate risk of bias, most often because of incomplete control for potential confounders, recruitment limited to hospital settings, or insufficient adjustment for clinical covariates. None of the studies reached the level of serious or critical risk. A breakdown of the judgments across individual domains for each study can be found in Supplementary Figures S1 and S2.

### 3.3. Meta-Analysis

**Figure 2:**
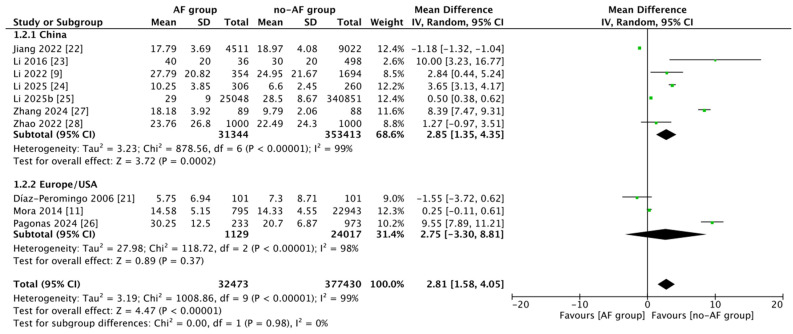
Forest plot of mean differences (MD, 95% CI) in lipoprotein(a) concentration between atrial fibrillation (AF) and non-AF groups. Subgroup analyses are presented for studies conducted in China [9,22,23,24,27,28] and in Europe/USA [11,21,25,26].

The overall effect showed that patients with AF had significantly higher Lp(a) concentrations (pooled mean difference = 2.81 mg/dL; 95% CI: 1.58–4.05; Z = 4.49; *p* < 0.0001) compared with controls (Figure 2). Nonetheless, the heterogeneity among studies was exceedingly high (I^2^ = 99%; τ^2^ = 3.19; χ^2^ = 1008.86, *p* < 0.00001), signifying considerable inconsistency in the magnitude of effects. In the subgroup analysis by geographical setting, studies conducted in China [9,22,23,25,27,28] exhibited a significant positive association (MD = 3.79; 95%CI: 0.15–7.43; I^2^ = 99%), and studies from Europe/USA [11,21,24,26] also demonstrated a statistically significant effect (MD = 2.04; 95%CI: 0.51–3.57; I^2^ = 97%).

### 3.4. Sensitivity Analysis

A leave-one-out (LOO) sensitivity analysis was performed to assess the robustness of the pooled effect size. In this method, each study was removed one at a time, and the random-effects model was recalculated. The pooled mean difference’s direction and statistical significance stayed the same through all iterations, with estimates ranging from 2.02 to 3.56. The most significant decline occurred after the removal of Zhang et al. [27] (pooled MD 2.02; Δμ = −0.84), succeeded by Pagonas et al. [26] (pooled MD 2.07; Δμ = −0.79). On the other hand, leaving out Jiang [22] (pooled MD 3.56; Δμ = +0.70) and Li et al. [25] (pooled MD 3.51; Δμ = +0.65) caused the most significant changes in the positive direction. Interestingly, no scenario saw the 95% confidence interval cross the null, suggesting that a single study did not influence the observed association.

**Figure 3:**
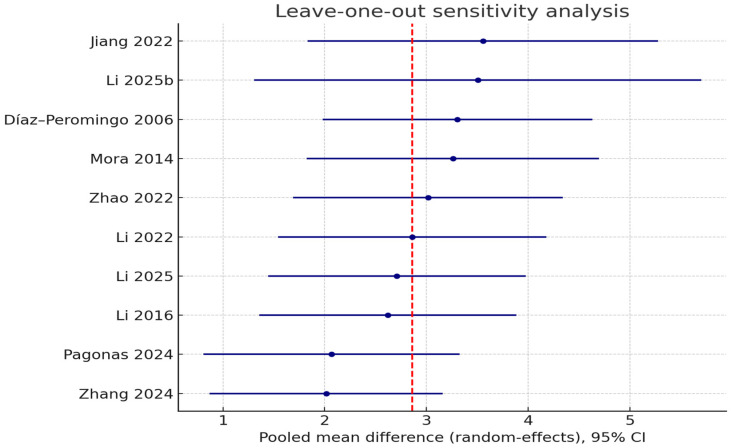
Leave-one-out sensitivity analysis of the association between lipid-related biomarkers and atrial fibrillation (AF). After removing one study at a time, each line shows the pooled mean difference (random-effects model) with 95% confidence intervals. The vertical dashed line shows the overall pooled estimate that includes all of the studies. The analysis shows that the overall association stayed the same, and no one study had an unfairly large effect on the summary effect [9,11,21,22,23,24,25,26,27,28].

Figure 3 shows the LOO forest-type plot, which shows the pooled estimates and confidence intervals that were found after each trial was removed one at a time. Figure S3 shows the Baujat diagnostic plot, which shows how each study affects the overall heterogeneity (*x*-axis) and the pooled effect size (*y*-axis). Both plots show that Zhang et al. [27] and Jiang et al. [22] had a big effect on the difference between studies, while Pagonas et al. [26] had a big effect on the pooled mean difference. Supplementary Table S3 contains the full results of the LOO sensitivity analysis.

### 3.5. Meta-Regression

In Model A, the biomarker type (HDL function/oxidation vs. Lp(a)) did not significantly predict the effect size (slope +2.45 MD, 95% CI −7.97 to +12.87; τ^2^ = 23.6). In Model B, with a significant decrease in τ^2^ to 10.2, population moderators demonstrated positive but non-significant associations: CAD +5.96 MD (95% CI −1.95 to +13.88) and HF +5.81 MD (95% CI −3.86 to +15.47). In Model C, while the biomarker type remained non-significant (slope +0.63 MD, 95% CI −10.02 to +11.28), the population indicators (CAD +5.87 MD; HF +5.50 MD) remained positive (combined); τ^2^ was 10.9. Although the large Hartung–Knapp intervals highlight the limited power given the small number of studies (k = 10), these results imply that the clinical population explains more heterogeneity than biomarker type alone. Supplementary Tables S4–S6 offer comprehensive coefficient estimates for every model.

### 3.6. Publication Bias Assessment

A visual examination of the funnel plot did not indicate significant asymmetry. Egger’s test (intercept = 6.65, *p* = 0.13: Figure S4) and Begg’s test (τ = 0.38, *p* = 0.16; Figure S5) did not indicate the presence of small-study effects. The trim-and-fill method did not add any new studies, and the bias-adjusted pooled effect (MD = 2.82) was almost the same as the first estimate. Rosenthal’s fail-safe N also showed that more than 1600 more null studies would be needed to make the overall result not significant, which shows how strong the association was. These analyses collectively indicate that publication bias is improbable to entirely elucidate the findings, although the conclusions necessitate cautious interpretation due to the restricted number of studies (k = 10) and considerable heterogeneity.

### 3.7. Certainty of Evidence

The GRADE method was used to look at the certainty of evidence in five areas: risk of bias, inconsistency, indirectness, imprecision, and publication bias (Table 2). The initial level of certainty was deemed low due to the observational nature of all included studies. The evidence was further downgraded because there was a high risk of bias (most studies were rated at moderate risk using ROBINS-E), a very high level of inconsistency (I^2^ = 99%), indirectness caused by different study populations and biomarker assessments, and imprecision shown by wide confidence intervals in several analyses. Formal statistical tests did not substantiate the existence of publication bias; however, the limited number of studies constrained the efficacy of these evaluations.

## 4. Discussion

Our pooled analysis revealed a statistically significant association between Lp(a) and AF; however, the certainty of the evidence was rated as very low according to the GRADE methodology. Rather than diminishing the importance of this finding, this highlights the urgent need to strengthen the evidence base through large, well-designed prospective studies and mechanistic investigations. Advancing this research agenda is essential to clarify causality, refine risk prediction, and ultimately inform targeted prevention and therapeutic strategies.

All studies included were observational, and the overall certainty was diminished due to significant heterogeneity, residual confounding, the indirectness of the populations and biomarkers evaluated, and the imprecision of certain effect estimates. It is essential to acknowledge that individual studies yielded inconsistent results concerning the direction of the association between Lp(a) and AF. For instance, one study [22] found that lower Lp(a) levels were linked to a higher risk of AF, while most other studies found a positive link. These differences likely elucidate the significant heterogeneity observed (I^2^ = 99%) and underscore the necessity for caution in interpreting the results and the imperative for more standardized research. Recognizing a very low certainty of evidence significantly enhances the credibility of our study by highlighting the necessity for caution in interpretation. It also points out important research gaps and stresses the need for large, high-quality prospective studies with standardized biomarker assessment and strict control for confounding. These studies are necessary to validate and enhance our findings, thereby augmenting the reliability of biomarker-based risk stratification in AF.

Our results augment previous meta-analyses regarding this subject. Yang et al. [30] indicated no significant correlation between Lp(a) and atrial fibrillation; however, their analysis was predicated on a limited number of studies featuring mixed observational and MR designs, and it did not include subgroup or sensitivity analyses. In contrast, Singh et al. [31] identified a modest positive genetic association in Mendelian randomization data predominantly involving European populations. Our current analysis, which combines ten observational studies from different groups, adds to these genetic findings by giving an empirical estimate of the differences in circulating Lp(a) levels between people with AF and those without, backed up by strong sensitivity and meta-regression analyses. These findings indicate that both genetic and phenotypic evidence suggest a connection between Lp(a) and AF; however, the relationship is heterogeneous and likely affected by ethnicity, comorbidity, and measurement techniques.

Lp(a) is a distinctive lipoprotein endowed with pro-atherogenic, pro-thrombotic, and pro-inflammatory properties [29,32,33]. Elevated circulating Lp(a) has been recognized as an independent risk factor for cardiovascular disease. Although Lp(a) is often described as an LDL-like particle, the risk attributable to higher Lp(a) levels is independent of that linked to LDL-C [34], implying divergent pathogenic pathways. Mechanistically, Lp(a) triggers monocyte-driven inflammation via its OxPL cargo, initiating leukocyte recruitment [35]. In parallel, OxPL-bound Lp(a) directly perturbs endothelial stability, reducing nitric-oxide bioavailability and increasing ROS, thereby sustaining vascular inflammation [36,37]. Contemporary models of AF stress that inflammation is tightly linked with both electrical and structural atrial remodeling [38,39,40]. Inflammatory mediators modulate ion-channel function and calcium homeostasis, alter action-potential duration, and disturb intracellular calcium handling, which are mediators that facilitate ectopic activity and re-entry circuits central to AF initiation and persistence. At the same time, inflammatory signaling activates fibroblasts, accelerates collagen deposition and atrial fibrosis, and promotes cardiomyocyte apoptosis, cumulatively reshaping atrial architecture [41]. Taken together, the inflammatory axis offers a coherent biological bridge linking higher circulating Lp(a), as a pro-inflammatory signal, to the development and progression of AF.

Moreover, Lp(a) has been associated with a heightened thrombotic propensity in patients with AF. A key structural feature, apolipoprotein(a), shares homology with plasminogen yet exerts different biological effects; by interfering with tissue plasminogen activator, apo(a) can reduce the conversion of plasminogen to plasmin [42], thereby attenuating fibrinolysis and favoring thrombus persistence. Lp(a) may also enhance coagulation by increasing platelet reactivity, upregulating tissue-factor expression, and inhibiting the tissue-factor pathway [43,44], collectively fostering a hypercoagulable milieu that coexists with systemic inflammation and atrial fibrosis features linked to adverse outcomes in AF [45]. Clinically, higher Lp(a) levels have been correlated with progression of atrial fibrosis and with disturbances in atrial structure and electrophysiology, potentially compounding the risk of intracardiac thrombus formation [33]. More broadly, accumulating observational data suggest AF is accompanied by a prothrombotic state characterized by multiple hematologic abnormalities, including elevated Lp(a) [46,47]. In line with this, Igarashi et al. reported an association between left-atrial thrombi in elderly patients with chronic AF and increased Lp(a), albeit in a small cohort [48]. Given Lp(a)’s combined anti-fibrinolytic and pro-coagulant effects in AF, therapeutic targeting of Lp(a) could be clinically meaningful. However, robust clinical trials and meta-analytic evidence specifically linking elevated Lp(a) to increased stroke risk within AF populations remain insufficient at present, underscoring a clear direction for future investigation.

Emerging evidence points to a role of epicardial adipose tissue (EAT) surrounding the atria in AF pathogenesis, acting via paracrine secretion of pro-inflammatory and pro-fibrotic mediators that drive atrial remodeling [49,50]. Quantitative characteristics of left-atrial EAT have also been associated with outcomes after catheter ablation [49,51]. Clinically, higher Lp(a) concentrations correlate with increased pericoronary adipose tissue inflammation on serial coronary computed tomography angiography (CTA) in a long-term prospective study [42,52]; consistent with this biology, 18F-FDG PET/CT demonstrates significantly higher arterial-wall glucose uptake in individuals with elevated Lp(a), indicating an activated inflammatory metabolic state [35]. These observations raise the hypothesis that a similar Lp(a)-inflammation axis may operate in periatrial EAT, amplifying fibro-inflammatory remodeling of the left atrium. This warrants integrated studies combining Lp(a) quantification with periatrial EAT phenotyping (e.g., volume, attenuation/radiomics) and AF outcomes.

Considering our results, we suggest that large prospective studies across multi-ethnic genetically diverse populations and geographical regions, with standardized Lp(a) assessment, are required. In parallel, translational investigations should clarify the mechanisms by which this genetically determined, diet-independent biomarker contributes to atrial fibrosis and remodeling. Another question is whether Lp(a)-lowering strategies can affect AF development and inhibit its progression. Such strategies could potentially have double benefit for patients who are at risk of cardiovascular disorders.

### Limitations

This meta-analysis has several limitations that merit caution. The between-study heterogeneity was extreme (I^2^ ≈ 99%), reflecting genuine differences in design, patient characteristics, and biomarker measurement; despite sensitivity checks and meta-regression, much of this variability remained unresolved. Most included reports were observational (prospective or retrospective cohorts) and hospital-based case–control/cross-sectional studies, so residual confounding, reverse causation, and uneven covariate adjustment (comorbidities, medications) are hard to exclude. Case ascertainment for atrial fibrillation also varied (ECG confirmation, health records, or self-report), and AF subtypes were seldom distinguished, limiting phenotype-specific conclusions. Most of the included studies defined AF based solely on its presence, without differentiating between paroxysmal, persistent, or permanent forms. Consequently, the impact of AF subtype on lp(a) levels could not be assessed, potentially contributing to residual heterogeneity. The evidence base leaned heavily toward Chinese cohorts, with only a large UK Biobank analysis and one U.S. female cohort, which narrows generalizability across populations. There was no consistency in the laboratory methods for lipoprotein(a), oxidized HDL, and related markers. Furthermore, the included studies used heterogeneous and often unreported assay methods for Lp(a) quantification. Most expressed concentrations as total mass (mg/dL) rather than particle number (nmol/L), which does not account for apo(a) isoform size heterogeneity. Given that Lp(a) levels are inversely correlated with the number of kringle IV type-2 repeats, this introduces potential bias and may contribute to between-study variability. Future studies should apply isoform-independent methods and standardized reporting to ensure comparability. Some studies used medians or non-standard units that needed to be converted, which could have led to measurement errors. Multiple datasets focused on high-risk clinical cohorts (e.g., CAD, chronic heart failure, hypertension), wherein underlying conditions and treatments may obscure associations. Finally, we synthesized study level (not individual participant data), which prevented harmonized adjustment, sex-stratified analyses, and exploration of dose–response or nonlinearity. Taken together, these issues suggest that the observed links between elevated Lp(a), lipoprotein dysfunction, and AF should be viewed as provisional and confirmed in large, multi-ethnic prospective cohorts using standardized assays and rigorous AF phenotyping.

## 5. Conclusions

Our meta-analysis revealed a statistically significant association between Lp(a) and AF; however, the certainty of the evidence was assessed as very low according to the GRADE methodology. This underscores the urgent need for large, multi-ethnic, geographically diverse prospective studies using standardized methods of Lp(a) assessment. Clarifying this association is critical for advancing mechanistic understanding, refining risk prediction, and informing the development of targeted preventive and therapeutic strategies.

## Figures and Tables

**Table 1:** Baseline characteristics of included studies.

Study	Country	Study Design	Study Group	Sample Size	Male (%)	Age	BMI	HTN (%)	DM2 (%)
Díaz-Peromingo et al., 2006 [21]	Spain	Case–control study	AF	101	NS	NS	NS	NS	NS
			Non-AF	101	NS	NS	NS	NS	NS
Jiang et al., 2022 [22]	China	Retrospective study	AF	4511	2388 (52.9%)	67.85 (44.95, 90.75)	23.62 (21.55, 25.78)	2505 (55.53%)	728 (16.14%)
			Non-AF	9022	4797 (53.2%)	67.85 (44.97, 90.73)	23.56 (21.45, 25.55)	5018 (55.62%)	1908 (21.14%)
Li et al., 2016 [23]	China	Prospective cohort study	AF	36	18 (50.0%)	71.9 (10.6)	NS	23 (63.9%)	20 (55.6%)
			Non-AF	498	300 (60.2%)	70.0 (14.6)	NS	301 (60.4%)	170 (34.1%)
Li et al., 2022 [9]	China	Observational case–control study	AF	354	65.69 (10.69)	202	24.15 (3.84)	209	83
			Non-AF	1694	63.89 (11.1)	1007	24.16 (3.97)	945	390
Li et al., 2025 [24]	China	Retrospective cohort study	AF	306	180 (58.8%)	71 (62, 77)	NS	NS	119 (38.9%)
			Non-AF	260	106 (40.8%)	66 (62, 76)	NS	NS	67 (25.8%)
Li et al., 2025 [25]	UK	Retrospective cohort study	AF	25,048	15,291 (61.1%)	64.0 (59.0, 66.0)	27.9 (25.3, 31.4)	11,289 (45.1%)	1401 (5.6%)
			Non-AF	340,851	150,016 (44.0%)	57.0 (49.0, 63.0)	26.7 (24.1, 29.6)	83,062 (24.4%)	9381 (2.8%)
Mora et al., 2014 [11]	USA	Prospective cohort study	AF	795	0 (0.0%)	58.7 (52.7, 65.0)	26.0 (23.2, 30.1)	323 (40.6%)	35 (4.4%)
			Non-AF	22,943	0 (0.0%)	52.6 (48.8, 58.3)	24.8 (22.3, 28.3)	5299 (23.1%)	528 (2.3%)
Pagonas et al., 2024 [26]	Germany	Cross-sectional observational multicenter study	AF	233	158 (67.8%)	73 (66, 77)	29 (26–32)	211 (90.6%)	77 (33.0%)
			Non-AF	973	624 (64.1%)	66 (57, 75)	28 (25, 32)	793 (81.5%)	256 (26.3%)
Zhang et al., 2024 [27]	China	Case–control study	AF	89	46 (51.7%)	68.2 (11.2)	NS	6 (6.7%)	51 (57.3%)
			Non-AF	88	44 (50.0%)	56.2 (13.8)	NS	2 (2.3%)	33 (37.5%)
Zhao et al., 2022 [28]	China	Case–control study	AF	1000	506 (50.6%)	70.45 (10.46)	NS	674 (67.4%)	306 (30.6%)
			Non-AF	1000	496 (49.6%)	69.80 (9.84)	NS	329 (32.9%)	160 (16.0%)

Legend: AF—atrial fibrillation; BMI—body mass index (kg/m2); HTN—hypertension; DM2—type 2 diabetes mellitus; NS—not specified.

**Table 2:** Summary of findings and certainty of evidence (GRADE).

No. of Studies	Study Design	Risk of Bias	Inconsistency	Indirectness	Imprecision	Other Considerations	Effect (95% CI)/ Certainty
10	Observational studies	Serious (ROBINS-E: mostly moderate)	Very serious (I^2^ = 99%)	Serious (heterogeneous populations/biomarkers)	Serious (wide CIs)	Potential publication bias	MD = 2.81 (95%CI: 1.58–4.05) Certainty: ⨁○○○ very low

Explanation: ⨁ and ○ symbols represent the certainty of evidence according to the GRADE system. Each filled circle (⨁) indicates one level of certainty, while empty circles (○) indicate levels not achieved. For example, ⨁○○○ corresponds to very low certainty.

## Data Availability

The data that support the findings of this study are available from the corresponding author upon reasonable request.

## Supplementary Materials

The following supporting information can be downloaded at: https://www.mdpi.com/article/10.3390/jcm14217770/s1, Table S1: PRISMA checklist; Table S2: Search strategy; Table S3: Sensitivity analysis; Table S4: Meta-regression results for biomarker type (Model A); Table S5: Meta-regression results for clinical population (Model B); Table S6: Combined meta-regression model (Model C); Figure S1: Risk of bias assessment across included studies using the ROBINS-E tool; Figure S2: Summary of risk of bias across studies using the ROBINS-E tool; Figure S3: Baujat plot assessing the contribution of individual studies to overall heterogeneity and their influence on the pooled effect estimate; Figure S4: Egger’s funnel plot of effect size (mean difference) versus standard error (SE) with regression overlay; Figure S5: Begg’s test plot of effect size (mean difference) versus standard error (SE).

## Abbreviations

The following abbreviations are used in this manuscript:

AF	Atrial fibrillation
apo(a)	Apolipoprotein(a)
CAD	Coronary artery disease
CI	Confidence interval
CTA	Computed tomography angiography
ECG	Electrocardiogram
EAT	Epicardial adipose tissue
GRADE	Grading of Recommendations, Assessment, Development, and Evaluation
HF	Heart failure
LDL	Low-density lipoprotein
LDL-C	Low-density lipoprotein cholesterol
LOO	Leave-one-out
Lp(a)	Lipoprotein(a)
MD	Mean difference
OxPLs	Oxidized phospholipids
PET/CT	Positron emission tomography/computed tomography
PRISMA	Preferred Reporting Items for Systematic Reviews and Meta-Analyses
PROSPERO	International Prospective Register of Systematic Reviews
ROBINS-E	Risk of Bias in Non-randomized Studies of Exposures
ROS	Reactive oxygen species
SD	Standard deviation
